# 2-Benzyl­isoindoline-1,3-dione: a monoclinic polymorph

**DOI:** 10.1107/S1600536807065336

**Published:** 2007-12-21

**Authors:** Zhou Jiang, Jun-Dong Wang, Nai-Sheng Chen, Jin-Ling Huang

**Affiliations:** a Institute of Research on Functional Materials, Department of Chemistry, University of FuZhou, Fuzhou 350002, People’s Republic of China

## Abstract

In the molecule of the title compound, C_15_H_11_NO_2_, the dihedral angle between the ring systems is 81.3 (2)°. In the crystal structure, mol­ecules are held together *via* C—H⋯O inter­actions.

## Related literature

For the crystal structure of the triclinic form, see: Warzecha, Lex & Griesbeck (2006 ▶). For related literature, see: Warzecha, Görner & Griesbeck (2006 ▶); Orzeszko *et al.* (2000 ▶).
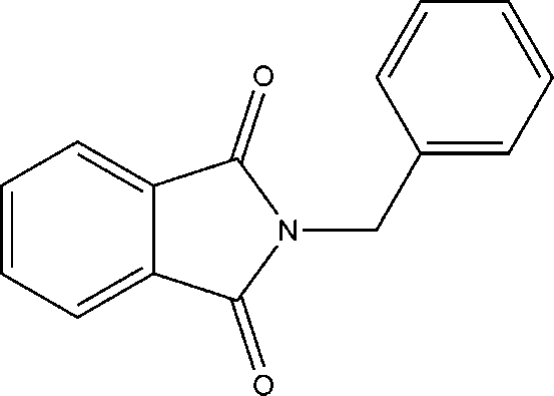


## Experimental

### 

#### Crystal data

C_15_H_11_NO_2_
*M* *_r_* = 237.25
Monoclinic, 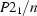

*a* = 8.8324 (6) Å
*b* = 5.3656 (4) Å
*c* = 25.1926 (18) Å
β = 98.851 (3)°
*V* = 1179.69 (15) Å^3^
*Z* = 4
Mo *K*α radiation
μ = 0.09 mm^−1^
*T* = 298 (2) K
0.8 × 0.2 × 0.1 mm

#### Data collection

Rigaku R-AXIS RAPID IP diffractometer
Absorption correction: none
3668 measured reflections
2083 independent reflections
1439 reflections with *I* > 2σ(*I*)
*R*_int_ = 0.048

#### Refinement

*R*[*F* ^2^ > 2σ(*F* ^2^)] = 0.097
*wR*(*F* ^2^) = 0.192
*S* = 1.26
2083 reflections
208 parameters
All H-atom parameters refined
Δρ_max_ = 0.24 e Å^−3^
Δρ_min_ = −0.19 e Å^−3^

### 

Data collection: *RAPID-AUTO* (Rigaku, 2006 ▶); cell refinement: *RAPID-AUTO*; data reduction: *RAPID-AUTO*; program(s) used to solve structure: *SHELXS97* (Sheldrick, 1997 ▶); program(s) used to refine structure: *SHELXL97* (Sheldrick, 1997 ▶); molecular graphics: *ORTEX* (McArdle, 1995 ▶); software used to prepare material for publication: *SHELXL97*.

## Supplementary Material

Crystal structure: contains datablocks I, global. DOI: 10.1107/S1600536807065336/tk2231sup1.cif

Structure factors: contains datablocks I. DOI: 10.1107/S1600536807065336/tk2231Isup2.hkl

Additional supplementary materials:  crystallographic information; 3D view; checkCIF report

## Figures and Tables

**Table 1:** Hydrogen-bond geometry (Å, °)

*D*—H⋯*A*	*D*—H	H⋯*A*	*D*⋯*A*	*D*—H⋯*A*
C15—H7⋯O1^i^	1.03 (6)	2.43 (6)	3.425 (6)	161 (5)
C3—H1⋯O2^ii^	0.98 (5)	2.54 (5)	3.363 (7)	142 (4)

Symmetry codes: (i) 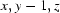; (ii) 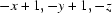.

**Fig. 1.**
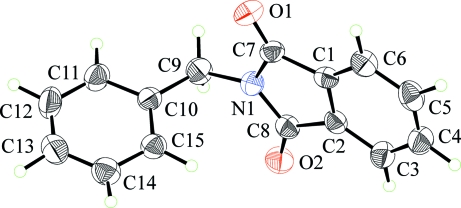
The molecular structure of (I) showing the atomic numbering scheme. Displacement ellipsoids are drawn at the 35% probability level.

## supplementary crystallographic information

### Comment

The title compound, *N*-Benzylphthalimide (2-benzylisoindoline-1,3-dione) (I), plays an important role in photoinduced electron transfer (PET) reactions (Warzecha, Görner & Griesbeck, 2006). Warzecha, Lex & Griesbeck (2006) also reported the crystal structure of the triclinic form of (I). Herein, the crystal structure of a monoclinic form is described.

The molecular structure of (I), Fig. 1, shows two planar subunits, *i.e.* a phthalimide moiety and a phenyl ring, being linked by a methylene-C9 atom with a N1—C9—C10 bond angle of 114.2 (5)°. The dihedral angle formed between the least-squares planes through each of the subunits is 81.3 (2)°. The C8—N1—C9—C10 and C7—N1—C9—C10 torsion angles of 91.3 (6)Å and -88.0 (6)°, respectively, highlight the orthogonal relationship within the molecule.

The crystal packing is stabilized by C—H···O interactions (Table 1).

### Experimental

Compound (I) was purified by silica-gel column chromatography with alcohol-hexane (*v*/*v* = 3/7) as eluent. Single crystals were obtained by slow evaporation of the eluting solution at room temperature.

### Refinement

The H atoms were refined: range of C—H = 0.91 (6) - 1.04 (6) Å.

### Crystal data

**Table:**

C_15_H_11_N_1_O_2_	*F*_000_ = 496
*M**_r_* = 237.25	*D*_x_ = 1.336 Mg m^−^^3^
Monoclinic, *P*2_1_/*n*	Mo *K*α radiation λ = 0.71069 Å
Hall symbol: -P 2yn	Cell parameters from 3962 reflections
*a* = 8.8324 (6) Å	θ = 1.2–25.0º
*b* = 5.3656 (4) Å	µ = 0.09 mm^−^^1^
*c* = 25.1926 (18) Å	*T* = 298 (2) K
β = 98.851 (3)º	Needle, colorless
*V* = 1179.69 (15) Å^3^	0.8 × 0.2 × 0.1 mm
*Z* = 4	

### Data collection

**Table:**

Rigaku R-AXIS RAPID IP diffractometer	1439 reflections with *I* > 2σ(*I*)
Radiation source: Rigaku rotating anode generator	*R*_int_ = 0.048
Monochromator: Graphite Monochromator	θ_max_ = 25.0º
*T* = 298(2) K	θ_min_ = 1.6º
ω scans	*h* = −10→9
Absorption correction: none	*k* = −6→6
3668 measured reflections	*l* = −29→13
2083 independent reflections	

### Refinement

**Table:**

Refinement on *F*^2^	Hydrogen site location: inferred from neighbouring sites
Least-squares matrix: full	All H-atom parameters refined
*R*[*F*^2^ > 2σ(*F*^2^)] = 0.097	*w* = 1/[σ^2^(*F*_o_^2^) + (0.0216*P*)^2^ + 2.0256*P*] where *P* = (*F*_o_^2^ + 2*F*_c_^2^)/3
*wR*(*F*^2^) = 0.192	(Δ/σ)_max_ < 0.001
*S* = 1.26	Δρ_max_ = 0.24 e Å^−^^3^
2083 reflections	Δρ_min_ = −0.19 e Å^−^^3^
208 parameters	Extinction correction: SHELXL97 (Sheldrick, 1997), Fc^*^=kFc[1+0.001xFc^2^λ^3^/sin(2θ)]^-1/4^
Primary atom site location: structure-invariant direct methods	Extinction coefficient: 0.008 (2)
Secondary atom site location: difference Fourier map	

### Special details

**Table:**

Experimental. collimator diameter: 0.800000 mm
Geometry. All e.s.d.'s (except the e.s.d. in the dihedral angle between two l.s. planes) are estimated using the full covariance matrix. The cell e.s.d.'s are taken into account individually in the estimation of e.s.d.'s in distances, angles and torsion angles; correlations between e.s.d.'s in cell parameters are only used when they are defined by crystal symmetry. An approximate (isotropic) treatment of cell e.s.d.'s is used for estimating e.s.d.'s involving l.s. planes.
Refinement. Refinement of *F*^2^ against ALL reflections. The weighted *R*-factor *wR* and goodness of fit *S* are based on *F*^2^, conventional *R*-factors *R* are based on *F*, with *F* set to zero for negative *F*^2^. The threshold expression of *F*^2^ > σ(*F*^2^) is used only for calculating *R*-factors(gt) *etc*. and is not relevant to the choice of reflections for refinement. *R*-factors based on *F*^2^ are statistically about twice as large as those based on *F*, and *R*- factors based on ALL data will be even larger.

### Fractional atomic coordinates and isotropic or equivalent isotropic displacement parameters (Å^2^)

**Table:**

	*x*	*y*	*z*	*U*_iso_*/*U*_eq_	
O2	0.5810 (4)	0.7318 (7)	0.08609 (13)	0.0656 (10)	
N1	0.7327 (4)	1.0824 (8)	0.09212 (14)	0.0520 (11)	
O1	0.8908 (4)	1.4018 (7)	0.07261 (13)	0.0679 (11)	
H1	0.579 (6)	0.595 (10)	−0.030 (2)	0.081*	
H5	0.631 (6)	1.076 (10)	0.1573 (18)	0.070 (15)*	
H10	1.040 (6)	1.130 (11)	0.309 (2)	0.085*	
H4	0.895 (6)	1.300 (11)	−0.042 (2)	0.085*	
H6	0.717 (7)	1.326 (13)	0.149 (2)	0.11 (2)*	
H11	0.834 (6)	1.292 (12)	0.244 (2)	0.10 (2)*	
H9	1.167 (6)	0.743 (11)	0.291 (2)	0.090 (18)*	
H2	0.666 (6)	0.677 (11)	−0.112 (2)	0.082 (17)*	
H3	0.815 (6)	1.037 (10)	−0.120 (2)	0.081 (16)*	
H8	1.099 (7)	0.588 (12)	0.204 (2)	0.10 (2)*	
H7	0.905 (7)	0.729 (12)	0.140 (2)	0.115*	
C1	0.7860 (5)	1.0946 (9)	0.00582 (17)	0.0476 (12)	
C2	0.6932 (5)	0.8927 (9)	0.00966 (17)	0.0483 (12)	
C3	0.6470 (6)	0.7357 (11)	−0.0330 (2)	0.0599 (14)	
C4	0.6984 (7)	0.7930 (12)	−0.0809 (2)	0.0670 (15)	
C5	0.7910 (6)	0.9958 (12)	−0.0849 (2)	0.0676 (16)	
C6	0.8377 (6)	1.1518 (11)	−0.0419 (2)	0.0600 (14)	
C7	0.8157 (5)	1.2218 (9)	0.05865 (18)	0.0495 (12)	
C8	0.6581 (5)	0.8804 (10)	0.06590 (18)	0.0509 (12)	
C9	0.7296 (7)	1.1484 (13)	0.1487 (2)	0.0620 (14)	
C10	0.8568 (5)	1.0365 (9)	0.18740 (17)	0.0495 (12)	
C11	0.8977 (7)	1.1438 (12)	0.2375 (2)	0.0652 (15)	
C12	1.0108 (8)	1.0414 (13)	0.2747 (2)	0.0787 (19)	
C13	1.0886 (7)	0.8336 (13)	0.2625 (2)	0.0744 (17)	
C14	1.0491 (7)	0.7241 (13)	0.2137 (2)	0.0711 (16)	
C15	0.9351 (6)	0.8245 (10)	0.1757 (2)	0.0581 (13)	

### Atomic displacement parameters (Å^2^)

**Table:**

	*U*^11^	*U*^22^	*U*^33^	*U*^12^	*U*^13^	*U*^23^
O2	0.069 (2)	0.063 (2)	0.066 (2)	−0.012 (2)	0.0146 (17)	0.0088 (19)
N1	0.058 (2)	0.052 (3)	0.046 (2)	0.001 (2)	0.0091 (18)	−0.002 (2)
O1	0.073 (2)	0.054 (2)	0.077 (2)	−0.012 (2)	0.0135 (18)	−0.015 (2)
C1	0.045 (2)	0.041 (3)	0.056 (3)	0.003 (2)	0.007 (2)	0.004 (2)
C2	0.048 (2)	0.049 (3)	0.047 (2)	0.008 (2)	0.0045 (19)	0.005 (2)
C3	0.058 (3)	0.064 (4)	0.056 (3)	−0.003 (3)	0.004 (2)	−0.002 (3)
C4	0.075 (4)	0.068 (4)	0.057 (3)	0.004 (3)	0.008 (3)	−0.008 (3)
C5	0.070 (3)	0.083 (4)	0.052 (3)	0.013 (3)	0.016 (3)	0.008 (3)
C6	0.058 (3)	0.064 (4)	0.060 (3)	0.000 (3)	0.017 (2)	0.006 (3)
C7	0.048 (3)	0.041 (3)	0.059 (3)	0.005 (2)	0.004 (2)	−0.004 (2)
C8	0.049 (3)	0.051 (3)	0.053 (3)	0.004 (3)	0.005 (2)	0.004 (3)
C9	0.069 (3)	0.064 (4)	0.055 (3)	0.010 (3)	0.017 (2)	−0.005 (3)
C10	0.060 (3)	0.047 (3)	0.045 (2)	−0.007 (3)	0.017 (2)	0.001 (2)
C11	0.080 (4)	0.065 (4)	0.052 (3)	−0.005 (3)	0.014 (3)	−0.008 (3)
C12	0.101 (5)	0.088 (5)	0.044 (3)	−0.017 (4)	0.002 (3)	−0.005 (3)
C13	0.074 (4)	0.080 (5)	0.067 (4)	−0.002 (4)	0.004 (3)	0.013 (4)
C14	0.072 (4)	0.067 (4)	0.074 (4)	0.007 (3)	0.011 (3)	0.003 (3)
C15	0.064 (3)	0.055 (3)	0.056 (3)	−0.001 (3)	0.012 (2)	−0.001 (3)

### Geometric parameters (Å, °)

**Table:**

O2—C8	1.210 (5)	C6—H4	0.94 (6)
N1—C8	1.383 (6)	C9—C10	1.496 (7)
N1—C7	1.413 (6)	C9—H5	1.01 (5)
N1—C9	1.473 (6)	C9—H6	0.96 (7)
O1—C7	1.193 (5)	C10—C11	1.383 (6)
C1—C2	1.371 (6)	C10—C15	1.386 (7)
C1—C6	1.384 (6)	C11—C12	1.376 (8)
C1—C7	1.483 (6)	C11—H11	1.00 (6)
C2—C3	1.377 (7)	C12—C13	1.370 (8)
C2—C8	1.498 (6)	C12—H10	0.99 (5)
C3—C4	1.388 (7)	C13—C14	1.359 (8)
C3—H1	0.98 (5)	C13—H9	1.04 (6)
C4—C5	1.374 (8)	C14—C15	1.385 (7)
C4—H2	1.00 (5)	C14—H8	0.91 (6)
C5—C6	1.382 (7)	C15—H7	1.03 (6)
C5—H3	0.97 (5)		
			
C8—N1—C7	112.5 (4)	N1—C8—C2	105.3 (4)
C8—N1—C9	124.9 (4)	N1—C9—C10	114.2 (4)
C7—N1—C9	122.6 (4)	N1—C9—H5	105 (3)
C2—C1—C6	121.1 (4)	C10—C9—H5	107 (3)
C2—C1—C7	109.0 (4)	N1—C9—H6	105 (4)
C6—C1—C7	129.8 (5)	C10—C9—H6	118 (4)
C1—C2—C3	122.5 (4)	H5—C9—H6	106 (5)
C1—C2—C8	108.3 (4)	C11—C10—C15	117.8 (5)
C3—C2—C8	129.2 (5)	C11—C10—C9	119.5 (5)
C2—C3—C4	116.5 (5)	C15—C10—C9	122.7 (4)
C2—C3—H1	122 (3)	C12—C11—C10	121.1 (6)
C4—C3—H1	122 (3)	C12—C11—H11	124 (3)
C5—C4—C3	121.1 (5)	C10—C11—H11	114 (3)
C5—C4—H2	123 (3)	C13—C12—C11	120.5 (6)
C3—C4—H2	116 (3)	C13—C12—H10	121 (3)
C4—C5—C6	122.1 (5)	C11—C12—H10	118 (3)
C4—C5—H3	117 (3)	C14—C13—C12	119.2 (6)
C6—C5—H3	120 (3)	C14—C13—H9	118 (3)
C5—C6—C1	116.7 (5)	C12—C13—H9	122 (3)
C5—C6—H4	127 (3)	C13—C14—C15	121.0 (6)
C1—C6—H4	116 (3)	C13—C14—H8	122 (4)
O1—C7—N1	124.6 (4)	C15—C14—H8	117 (4)
O1—C7—C1	130.6 (5)	C14—C15—C10	120.4 (5)
N1—C7—C1	104.8 (4)	C14—C15—H7	118 (3)
O2—C8—N1	125.0 (4)	C10—C15—H7	122 (3)
O2—C8—C2	129.7 (5)		

### Hydrogen-bond geometry (Å, °)

**Table:**

*D*—H···*A*	*D*—H	H···*A*	*D*···*A*	*D*—H···*A*
C15—H7···O1^i^	1.03 (6)	2.43 (6)	3.425 (6)	161 (5)
C3—H1···O2^ii^	0.98 (5)	2.54 (5)	3.363 (7)	142 (4)

Symmetry codes: (i) *x*, *y*−1, *z*; (ii) −*x*+1, −*y*+1, −*z*.
